# Steam Coating-Based Synthesis and Corrosion Inhibition Performance of Mg–Al-Layered Double Hydroxide Films with Different Interlayer Anions on Al-Si-Cu Alloys

**DOI:** 10.3390/ma18235405

**Published:** 2025-11-30

**Authors:** Io Matsui, Hikari Ouchi, Yuki Atsuumi, Kota Fukuhara, Takahiro Ishizaki

**Affiliations:** 1 Materials Science and Engineering, Graduate School of Engineering and Science, Shibaura Institute of Technology, Tokyo 135-8548, Japan; 2 College of Engineering, Shibaura Institute of Technology, Tokyo 135-8548, Japan

**Keywords:** Al–Si–Cu alloy, layered double hydroxide, steam coating process, corrosion resistance

## Abstract

Al–Si–Cu alloy is one of the aluminum die-cast alloys widely used in industry. Due to the presence of Si and Cu elements in the Al–Si–Cu alloy, the corrosion resistance of the Al–Si–Cu alloy is lowered. Thus, developing a corrosion-resistant film on the Al–Si–Cu alloy is necessary. A layered double hydroxide (LDH) film is recognized as a promising corrosion-resistant coating. LDHs exhibit a distinct structure where positively charged basic layers (metal hydroxides) are interleaved with intermediate layers that accommodate charge-compensating anions and hydration water. The positively charged layers allow for the exchange of anions as interlayers, enabling the incorporation of various anions into the interlayer. The difference in the anion species in the interlayer of the LDH films can affect corrosion-resistant performance. In this study, we aimed to prepare Mg–Al LDH films intercalated with different anions (NO_3_^−^, MoO_4_^2−^, VO_4_^3−^, and PO_4_^3−^) and investigate the corrosion resistance of the LDH films. The films were prepared on die-cast Al–Si–Cu alloys using steam coating and immersion processes. The prepared LDH films were characterized by XRD, SEM, FT-IR, and electrochemical measurements. The electrochemical measurements revealed that Mg–Al LDH films intercalated with MoO_4_^2−^ showed the most superior corrosion resistance among all films prepared in this study.

## 1. Introduction

Aluminum is the most abundant metallic element, constituting approximately 7.73% of the Earth’s crust. Due to its high affinity for oxygen, aluminum readily undergoes self-passivation by forming a stable oxide film on its surface [1,2]. As a result, aluminum generally demonstrates good corrosion resistance under natural environmental conditions. However, from a practical point of view, various alloying elements are added to aluminum to enhance specific properties, which have been widely developed and utilized. Aluminum alloys offer excellent thermal and electrical conductivity, ductility, and workability, making them suitable for various industrial applications, including transportation, electronics, aerospace, chemical processing, pharmaceuticals, and food production [3]. Die-cast aluminum alloys, in particular, are favored for their cost-effectiveness and high productivity in producing various industrial products, such as automobile components. Nonetheless, the alloying elements added to improve casting performance, such as Si and Cu, can adversely affect the corrosion resistance of aluminum alloys [4]. Furthermore, the corrosion resistance of Al–Si–Cu alloys is significantly compromised in environments containing aggressive corrosive species like chloride ions. Therefore, developing and applying corrosion-resistant films on the surfaces of Al–Si–Cu alloys is essential to enhance their durability in such harsh conditions [5,6].

Layered double hydroxide (LDH) films have been utilized to enhance the corrosion resistance of various alloys [7,8]. LDH is represented by the general formula [M^2+^_1−x_M^3+^_x_(OH)_2_]^x+^[A^n−^_x/n_]^x−^·mH_2_O (0.17 ≦ x ≦ 0.33), where M^2+^, M^3+^, and A^n−^ represent divalent metal ions (Mg^2+^, Zn^2+^, Cu^2+^, and Mn^2+^), trivalent metal ions (Al^3+^, Co^3+^, and Fe^3+^), and *n*-valent anions (NO_3_^−^, Cl^−^, CO_3_^2−^, and PO_4_^3−^), respectively [7,8,9]. An LDH possesses a structure like brucite (Mg(OH)_2_), characterized by alternating octahedral basic layers and intermediate layers composed of anions and interlayer water. The basic layer consists of double hydroxides in which trivalent cations replace a fraction of the divalent metals, resulting in a positively charged layer [10,11]. For Mg–Al LDH, the base layer is formed by the isomorphous substitution of Al^3+^ ions for some of the Mg^2+^ ions within the Mg(OH)_2_ host structure. LDH can exchange the anions in its intermediate layer through a process known as intercalation. This exchange is more favorable at higher charge densities and valences, making it likely for anions with higher charge densities and valences to be exchanged and retained within the LDH interlayer [12,13,14]. LDH films intercalated with various anions have been developed because the corrosion resistance of these films can vary based on the type of anion species present in the intermediate layer [15,16,17]. To further enhance the corrosion resistance of LDH films, certain anionic species that facilitate a self-healing function have been explored [18,19,20]. It has been reported that LDH intercalated with MoO_4_^2−^ and VO_4_^3−^ exhibited self-healing capabilities [18,19]. LDH containing PO_4_^3−^ has garnered significant attention due to its potential for self-repair [20]. It is believed that the type of anion species present in the interlayer can influence the self-healing ability and corrosion resistance of LDH. However, the effects of the specific anion species in improving the corrosion resistance of Al–Si–Cu alloys using LDH films have not yet been fully elucidated.

In this study, Mg–Al LDH films containing NO_3_^−^, MoO_4_^2−^, VO_4_^3−^, and PO_4_^3−^ as intermediate layers were prepared on die-cast Al–Si–Cu alloys using steam coating and immersion processes. The characteristics of the prepared films were evaluated through various methods, including X-ray diffraction (XRD), field-emission scanning electron microscopy (FESEM), FESEM energy dispersive X-ray spectroscopy (EDS), and Fourier-transform infrared spectroscopy (FT-IR). Furthermore, the corrosion resistance of the Mg–Al LDH films, incorporating NO_3_^−^, MoO_4_^2−^, VO_4_^3−^, and PO_4_^3−^ into the intermediate layer, was assessed through potentiodynamic polarization measurements and electrochemical impedance spectroscopy (EIS) in a 5.0 wt.% NaCl solution.

## 2. Materials and Methods

An Al–Si–Cu alloy (ADC12) with dimensions of 20 × 20 × 2 mm was used as the substrate. The atomic composition is detailed in Table 1.

As a pretreatment, the substrate surface was polished using #400, #1200, and #2000 waterproof abrasive paper, followed by ultrasonic cleaning (42 kHz) in ethanol (purity: 99.5%) for 10 min and then drying it using nitrogen gas (purity: 99.5%). Ultrapure water with an electrical resistivity of 18.2 MΩ·cm was served as the steam source. A total of 20 mL of ultrapure water was poured into a Teflon-lined autoclave (HU-100: SAN-AI KAGAKU Co., Ltd., Nagoya, Japan), and the cleaned substrate was placed on a sample stage within the autoclave. Next, 500 µL of a 1 M Mg(NO_3_)_2_·6H_2_O aqueous solution was dropped on the cleaned substrate, and then the autoclave was sealed tightly with a lid. The sealed autoclave was maintained in an electric furnace at 140 °C for 12 h to prepare a nitrate acid–type Mg–Al LDH film on the ADC12 substrate. Then, 10 mL of 1.0 M Na_2_MoO_4_·2H_2_O, 1.0 M Na_3_VO_4_, or 1.0 M H_3_PO_4_ aqueous solution, adjusted to pH 7, was prepared. The nitrate acid–type Mg–Al LDH films-coated substrates were immersed in the 1.0 M Na_2_MoO_4_·2H_2_O, Na_3_VO_4_, or H_3_PO_4_ aqueous solution and maintained for 1 h at room temperature to prepare MoO_4_^2−^, VO_4_^3−^, PO_4_^3−^-intercalated Mg–Al LDH films.

The crystalline phase of each film was identified from XRD patterns obtained through the 2θ method at an incident angle of 1° using a CuKα XRD device (Ultima IV, Rigaku Corp., Tokyo, Japan; 40 kV, 40 mA). Surface and cross-sectional observations, as well as elemental analysis, were conducted using field emission scanning electron microscopy with energy dispersive spectrometry (FESEM-EDS: JSM-IT300HR, JEOL Ltd., Tokyo, Japan; 30 kV). A cross-section of each film was prepared using a cross-section polisher (IB-09010CP, JEOL Ltd.) at an acceleration voltage of 6 kV for 7 h. The chemical bonding states of the films were examined through FT-IR (IRTracer-100, Shimadzu Co., Kyoto, Japan) to analyze their attenuated total reflection spectra. Their corrosion resistance was investigated through electrochemical measurements. All electrochemical experiments were conducted in a 5.0 wt.% NaCl aqueous solution (pH = 6.5) at room temperature using a computer-controlled potentiostat (VersaSTAT4, Princeton Applied Research, Ametek, Berwyn, PA, USA) under open-circuit conditions. The film-coated ADC12 substrates, produced through steam coating and immersion processes, served as the working electrodes, while a platinum mesh and saturated Ag/AgCl electrodes acted as the counter and reference electrodes, respectively. The corrosion potential of the specimens was measured by submerging them for 30 min in a 5.0 wt.% NaCl aqueous solution that was deaerated with nitrogen gas for 20 min. Afterward, a polarization curve was obtained at a scan rate of 0.167 mV/s and a potential scan range of −200 to +1200 mV relative to the corrosion potential. The nitrogen gas kept flowing over the liquid surface of the electrolytic cell during measurement. Electrochemical impedance spectroscopy (EIS) was performed to evaluate the corrosion resistance of the prepared films within a frequency range of 10 mHz to 100 kHz, using an amplitude of 5 mV. The experimental EIS spectra were analyzed based on equivalent electrical models using Zplot2.0 software to derive the fitting parameters.

## 3. Results

### 3.1. Characterization of LDH Films

**Figure 1:**
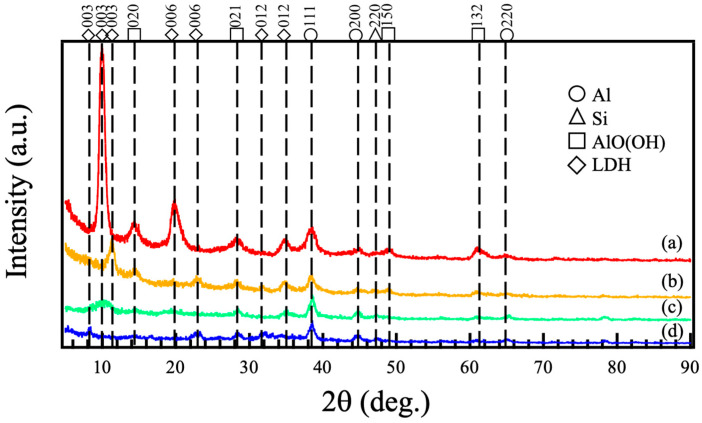
XRD patterns of films prepared on ADC12 substrates via (a) steam coating at 140 °C for 12 h using 1.0 M Mg(NO_3_)_2_ aqueous solution, followed by immersion in 1.0 M aqueous solutions of (b) Na_2_MoO_4_·2H_2_O, (c) Na_3_VO_4,_ and (d) H_3_PO_4_.

Figure 1 shows the XRD patterns of films prepared on ADC12 substrates via (a) steam coating at 140 °C for 12 h using 1.0 M Mg(NO_3_)_2_ aqueous solution, followed by immersion in 1.0 M aqueous solutions of (b) Na_2_MoO_4_·2H_2_O, (c) Na_3_VO_4_, and (d) H_3_PO_4_. The XRD pattern in Figure 1 (a) showed three diffraction peaks around 2θ = 10°, 20°, and 35°, corresponding to the 003, 006, and 012 reflections of nitrate-type Mg–Al LDH. Additionally, some diffraction lines were also observed at nearly 2θ = 14°, 28°, 49°, and 61°. These peaks are assigned to the diffraction lines attributed to the 020, 021, 150, and 132 reflections of AlO(OH). These findings demonstrate that crystalline Mg–Al LDH and AlO(OH) were formed on the ADC12 substrate via steam coating. The XRD patterns after immersion in different aqueous solutions (Figure 1 (b–d)) displayed diffraction peaks around 2θ = 8–12°, 24°, and 32–35°. These peaks also align with the 003, 006, and 012 reflections of Mg–Al LDH. Compared to the LDH before immersion, the intensity of the peak corresponding to the 003 reflection decreased significantly, and the peak position shifted. This suggests a change in the interlayer distance of the LDH. In the nitrate-type Mg–Al LDH, nitrate ions are likely present in the interlayer. Conversely, in the cases involving immersion in 1.0 M Na_2_MoO_4_·2H_2_O, 1.0 M Na_3_VO_4_, or 1.0 M H_3_PO_4_ aqueous solution, it is probable that MoO_4_^2−^, VO_4_^3−^, or PO_4_^3−^ ions are present in the interlayer, indicating a change in interlayer distance. The interlayer distances calculated from the 003 peaks of LDH in Figure 1 (a), (b), (c), and (d) are estimated to be 86.9 nm, 75.3 nm, 84.3 nm, and 107.3 nm, respectively. NO_3_^−^ has a planar structure, while MoO_4_^2−^, VO_4_^3−^, and PO_4_^3−^ are tetrahedral structures. The volumes of NO_3_^−^, MoO_4_^2−^, VO_4_^3−^, and PO_4_^3−^ have been reported to be 0.049, 0.088, 0.066, and 0.057 nm^3^, respectively [21,22]. While the intercalation of MoO_4_^2−^, VO_4_^3−^, and PO_4_^3−^ ions with volumes greater than NO_3_^−^ was expected to enlarge the interlayer space, the interlayer distances for MoO_4_^2−^ and VO_4_^3−^ surprisingly exhibited a decrease. This is likely a consequence of bridging coordination, leading to a reduction in the interlayer distance [23]. The XRD patterns in Figure 1 (b–d) also show diffraction lines at approximately 2θ = 14°, 28°, 49°, and 61°, corresponding to 020, 021, 150, and 132 reflections of AlO(OH). These results suggest that the steam coating produced a mixed film of nitrate-type Mg–Al LDH and AlO(OH), and subsequent immersion created a mixed film of Mg–Al LDH intercalated with MoO_4_^2−^, VO_4_^3−^, or PO_4_^3−^ along with AlO(OH).

### 3.2. Surface and Cross-Sectional Morphologies of Mg–Al LDH Films

**Figure 2:**
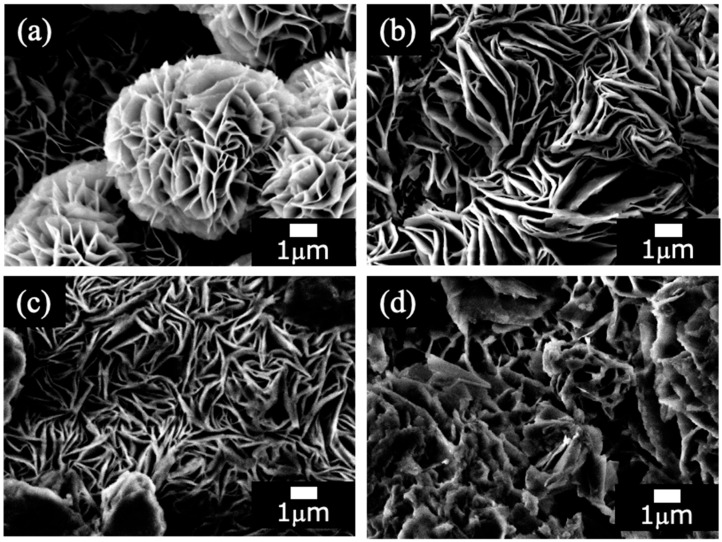
SEM images of films prepared on ADC12 substrates via (**a**) steam coating at 140 °C for 12 h using 1.0 M Mg(NO_3_)_2_ aqueous solution, followed by immersion in 1.0 M aqueous solutions of (**b**) Na_2_MoO_4_·2H_2_O, (**c**) Na_3_VO_4,_ and (**d**) H_3_PO_4_.

Figure 2 shows SEM images of the films prepared on ADC12 substrates via (Figure 2a) steam coating at 140 °C for 12 h using 1.0 M Mg(NO_3_)_2_ aqueous solution, followed by immersion in 1.0 M aqueous solutions of (Figure 2b) Na_2_MoO_4_·2H_2_O, (Figure 2c) Na_3_VO_4,_ and (Figure 2d) H_3_PO_4_. The SEM image of Figure 2a shows a petal-like structure composed of platelets with several µm in size, which is reported to be a characteristic structure of LDH [24]. This film (Figure 2a) exhibits a highly regular and distinct hierarchical structure (nanosheets to microspheres), appearing to grow radially from the center of the sphere. Figure 2b reveals a dense structure composed of randomly overlapping nanosheet layers, with no spherical aggregates observed. The individual sheets appear larger and thicker than those in Figure 2a. Similarly to Figure 2b, Figure 2c is primarily composed of overlapping nanosheets, but they are smaller and form a less dense, finer structure compared to Figure 2b. This morphology results in numerous gaps between the sheets, creating a fluffy structure where relatively thin sheets are stacked in a non-uniform manner. Figure 2d shows a structure where the nanosheet morphology is indistinct, having changed into a dense, bulk-like (blocky) structure. This transformation likely occurred due to the collapse and densification of the layered structure, possibly through processes such as dissolution and reprecipitation of the crystallites, or by the nanosheets increasing in thickness to become plate-like, resulting in a film that approaches a macroscopic bulk state. These structural differences could be the result of various parameters—such as the type of anions used (NO_3_^−^, MoO_4_^2−^, VO_4_^3−^, or PO_4_^3−^, or their intercalated ratio)—influencing the nucleation and growth processes of the film. The elemental composition of all films, determined by EDS, is listed in Table 2. From Table 2 (a), the presence of N was confirmed in the Mg–Al LDH prepared by steam coating at 140 °C for 12 h, suggesting that NO_3_^−^ could exist in the Mg–Al LDH film.

Following immersion of the Mg-Al LDH film in 1.0 M Na_2_MoO_4_ ·2H_2_O, 1.0 M Na_3_VO_4_, or 1.0 M H_3_PO_4_ aqueous solutions, the incorporation of Mo, V, and P was confirmed, respectively. However, the Mg-Al LDH film immersed in the Na_3_VO_4_ solution also retained N. This observation suggests that the anion exchange between NO_3_^−^ and VO_4_^3−^ may not have proceeded to a significant extent in these films due to charge balance and steric hindrance.

**Figure 3:**
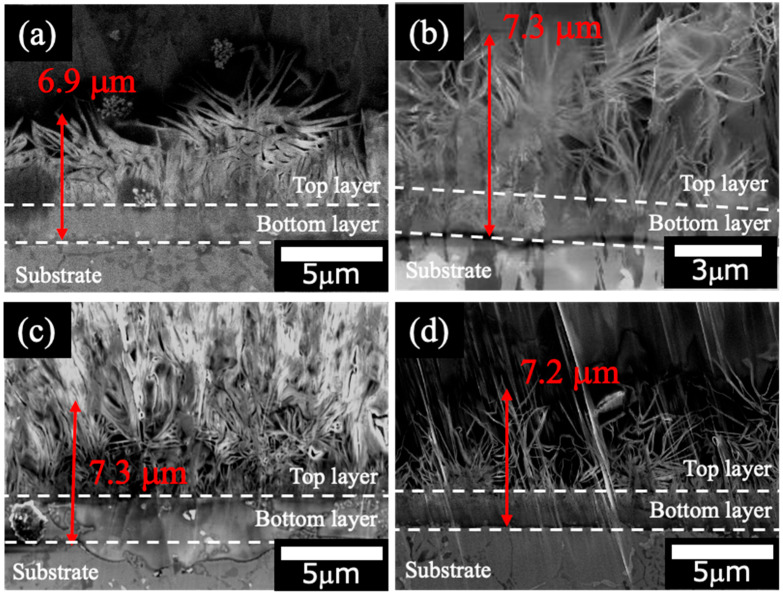
Cross-sectional SEM images of films prepared on ADC12 substrates via (**a**) steam coating at 140 °C for 12 h using 1.0 M Mg(NO_3_)_2_ aqueous solution, followed by immersion in 1.0 M aqueous solutions of (**b**) Na_2_MoO_4_·2H_2_O, (**c**) Na_3_VO_4,_ and (**d**) H_3_PO_4_.

Figure 3 shows the cross-sectional SEM images of the films prepared on ADC12 substrates via (Figure 3a) steam coating at 140 °C for 12 h using 1.0 M Mg(NO_3_)_2_ aqueous solution, followed by immersion in 1.0 M aqueous solutions of (Figure 3b) Na_2_MoO_4_·2H_2_O, (Figure 3c) Na_3_VO_4,_ and (Figure 3d) H_3_PO_4_. The thickness of all films was estimated to be (Figure 3a) 6.9 μm, (Figure 3b) 7.3 μm, (Figure 3c) 7.3 μm, and (Figure 3d) 7.2 μm, respectively. The films exhibited no significant change in thickness before and after immersion. This finding suggests that immersion in the Na_2_MoO_4_·2H_2_O, Na_3_VO_4,_ and H_3_PO_4_ solutions did not substantially alter the morphological integrity or structural dimensions of the films. The cross-sectional SEM images revealed that all films exhibited a double-layer structure. The bottom layer demonstrated a dense morphology, whereas the top layer contained gaps within its layered structure. A plate-like morphology was evident in the top layer, consistent with the SEM images shown in Figure 2. The SEM and XRD results conclusively identify the top layer as LDH and the underlying layer as AlO(OH).

### 3.3. Chemical Bonding States of Films

**Figure 4:**
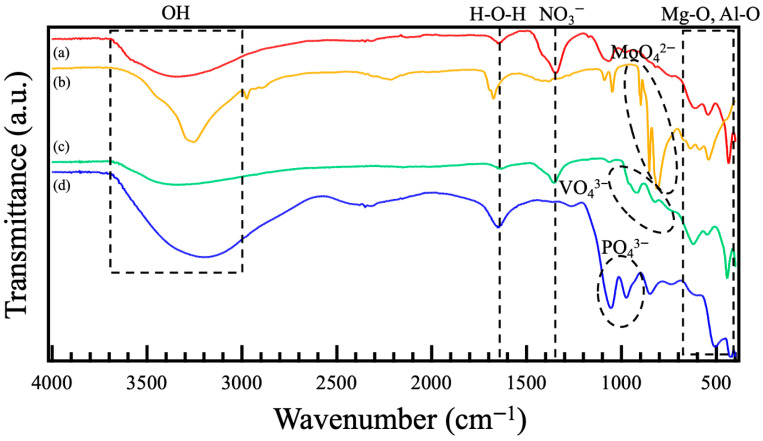
FT-IR spectra of films prepared on ADC12 substrates via (a) steam coating at 140 °C for 12 h using 1.0 M Mg(NO_3_)_2_ aqueous solution, followed by immersion in 1.0 M aqueous solutions of (b) Na_2_MoO_4_·2H_2_O, (c) Na_3_VO_4,_ and (d) H_3_PO_4_.

Figure 4 shows FT-IR spectra of the films prepared on ADC12 substrates via (a) steam coating at 140 °C for 12 h using 1.0 M Mg(NO_3_)_2_ aqueous solution, followed by immersion in 1.0 M aqueous solutions of (b) Na_2_MoO_4_·2H_2_O, (c) Na_3_VO_4,_ and (d) H_3_PO_4_. In Figure 4 (a), two absorption peaks assigned to Al–O lattice vibration and symmetric stretching vibrations of octahedral AlO_6_ are observed at around 420 cm^−1^ and 600 cm^−1^, respectively [25]. In addition, two peaks attributed to Mg–O or Al–O lattice vibration were also observed at around 450 cm^−1^ and 550 cm^−1^ [26]. Absorption peaks assigned to N–O were also observed at 1350 cm^−1^, and a broad peak related to interlayer water was present at 3450 cm^−1^ [27]. These results suggest that Mg–Al LDH, including NO_3_^−^ between interlayers, was formed. In the FT-IR spectra of the films immersed in the different aqueous solutions, each peak derived from Mo–O, V–O, and P–O was observed at around 800~900 cm^−1^ in Figure 4 (b), 800~1000 cm^−1^ in Figure 4 (c), and 900~1100 cm^−1^ in Figure 4 (d), respectively [28,29,30,31]. By the immersion of the nitrate-type Mg–Al LDH films prepared by steam coating in 1.0 M Na_2_MoO_4_·2H_2_O, 1.0 M Na_3_VO_4_, and 1.0 M H_3_PO_4_ aqueous solutions, the peak intensity attributed to N–O at around 1350 cm^−1^ was lowered, and the peaks assigned to Mo–O at around 800~900 cm^−1^, V–O at around 800~1000 cm^−1^, and P–O at around 900~1100 cm^−1^, respectively, appeared, indicating the occurrence of intercalation of MoO_4_^2−^, VO_4_^3−^, and PO_4_^3−^ with NO_3_^−^. However, in the FT-IR spectra of the films intercalated with Na_3_VO_4_ solution, the peak attributed to N–O at around 1350 cm^−1^ and the weak peak originating from V–O at around 800~1000 cm^−1^ remained. This indicates that the rate for the intercalation of VO_4_^3−^ with NO_3_^−^ could be low. This result agrees well with the results of elemental analysis by EDS in Table 2 (c), where N remained in the films after the immersion in the Na_3_VO_4_ aqueous solution. Therefore, it is inferred that the pH of the Na_3_VO_4_ aqueous solution for the intercalation was adjusted to 7, so a part of VO_4_^3−^ became protonated to HVO_4_^2−^ and its ionic radius and charge density changed, making it difficult for the introduction of the HVO_4_^2−^ into the interlayer to occur.

### 3.4. Corrosion Resistance of LDH Films

**Figure 5:**
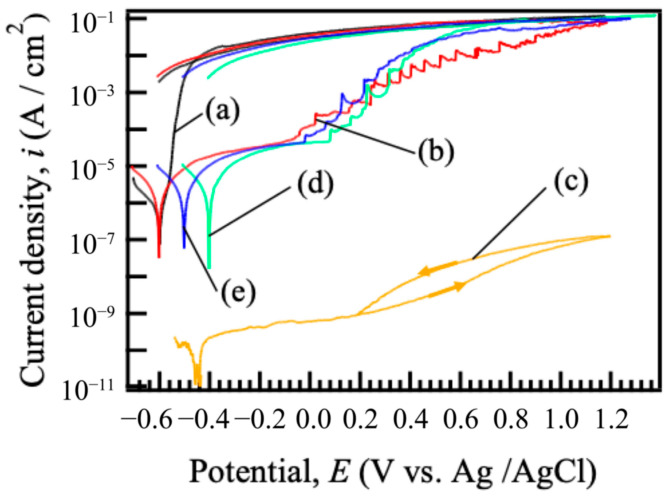
Cyclic potentiodynamic polarization curves of (a) bare ADC12 and films prepared on ADC12 substrates via (b) steam coating at 140 °C for 12 h using 1.0 M Mg(NO_3_)_2_ aqueous solution, followed by immersion in 1.0 M aqueous solutions of (c) Na_2_MoO_4_·2H_2_O, (d) Na_3_VO_4_, and (e) H_3_PO_4_.

Figure 5 shows the cyclic potentiodynamic polarization curves of (a) bare ADC12 and films prepared on ADC12 substrates via (b) steam coating at 140 °C for 12 h using 1.0 M Mg(NO_3_)_2_ aqueous solution, followed by immersion in 1.0 M aqueous solutions of (c) Na_2_MoO_4_·2H_2_O, (d) Na_3_VO_4_, and (e) H_3_PO_4_. The corrosion potential, *E*_corr_, and corrosion current density, *i*_corr_, values of untreated ADC12 were found to be –0.60 V and 1.34 × 10^−6^ A/cm^2^, respectively. When the potential was swept in the anodic direction from *E*_corr_, the current density increased rapidly, and the increase in the current density continued until approximately −0.40 V. Then, the current density exhibited a slower increase and approached a constant value. This behavior can be attributed to the dissolution of the substrate surface and the formation of a protective film. The increase in current density was limited when the potential was shifted to a more positive direction due to active aluminum dissolution nearing the diffusion limit. *E*_corr_ and *i*_corr_ values of nitrate-type Mg–Al LDH were found to be –0.60 V and 5.82 × 10^−6^ A/cm^2^, respectively. When the current density was adjusted positively above *E*_corr_, a gentle increase in current density was observed up to around –0.08 V. Compared to untreated ADC12, the anodic current density was reduced considerably, indicating improved corrosion resistance due to the preparation of the nitrate-type Mg–Al LDH film. Furthermore, the polarization curve of (b) nitrate-type Mg-Al LDH showed a rapid rise and fall in current density at the potentials of –0.08 to 0.80 V, suggesting that pitting corrosion and re-passivation are occurring. The pitting potential, *E*_pit_, value of the nitrate-type Mg-Al LDH was found to be –0.08 V. The *E*_corr_ and *i*_corr_ values of the Mg–Al LDH films after the immersion in 1.0 M Na_2_MoO_4_·2H_2_O, Na_3_VO_4_, and H_3_PO_4_ solutions were found to be –0.44, –0.39, and –0.49 V, and 2.58 × 10^−10^, 5.92 × 10^−6^, and 6.46 × 10^−6^ A/cm^2^, respectively. Compared to the untreated ADC12, all *E*_corr_ values for film-coated ADC12 were found to be more positive. In addition, comparison of the nitrate-type Mg–Al LDH prepared at 140 °C for 12 h and the Mg–Al LDH films after the immersion in 1.0 M Na_2_MoO_4_·2H_2_O, Na_3_VO_4_, and H_3_PO_4_ solutions showed that the intercalated films had lower current density and more positive *E*_corr_. Similarly to the trend observed for film (b), films (d) and (e) displayed a distinct sharp rise and fall in current density in the anodic region. The *E*_pit_ values of the Mg–Al LDH films after the immersion in 1.0 M Na_3_VO_4_ and H_3_PO_4_ solutions were found to be 0.06 and 0.02 V, respectively. In contrast, the film (c) did not exhibit a similar increase in current density and showed passive behavior. This behavior is hypothesized to be unique to the film intercalated with MoO_4_^2−^ because this anion demonstrates self-healing capability. In contrast, for the LDH films intercalated with NO_3_^−^, VO_4_^3−^, and PO_4_^3−^, the absence of self-healing action is presumed to result in the observed increase and subsequent decrease in current density, likely driven by the dissolution and reprecipitation of the film. In contrast, the MoO_4_^2−^-intercalated film showed a decrease in current density. This variance can be primarily attributed to the influence of the interlayer anions on the cathodic reaction kinetics, specifically the Oxygen Reduction Reaction (ORR) and the Hydrogen Evolution Reaction (HER) activity. MoO_4_^2−^ is considered to undergo reversible reduction to MoO_3_, which can form a highly protective layer that suppresses the cathodic reaction (ORR and HER). Comparing the corrosion current density of the intercalated Mg–Al LDH films, the values decreased in the order of MoO_4_^2−^, VO_4_^3−^, and PO_4_^3−^. These results indicate that the corrosion resistance of the Mg-Al LDH films, which is influenced by the intercalated anions, increased in the order of MoO_4_^2−^, VO_4_^3−^, and PO_4_^3−^.

**Figure 6:**
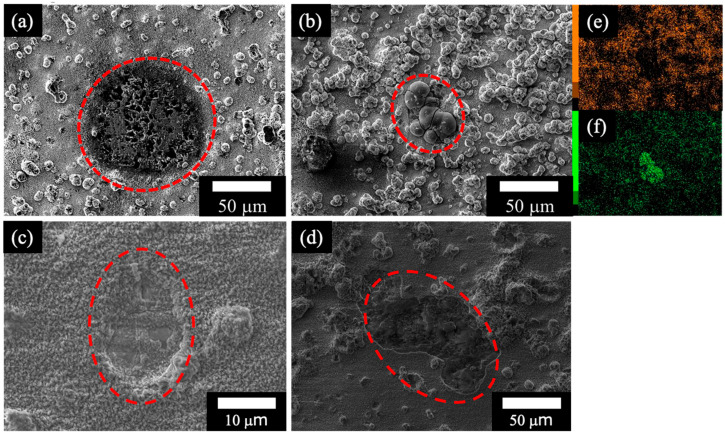
SEM images of LDH film prepared by (**a**) steam coating at 140 °C for 12 h using 1.0 M Mg(NO_3_)_2_ aqueous solution, followed by immersion in 1.0 M aqueous solutions of (**b**) Na_2_MoO_4_·2H_2_O, (**c**) Na_3_VO_4,_ and (**d**) H_3_PO_4_ after polarization test. Elemental mapping images of the defect part on the film: (**e**) Mg and (**f**) Mo. The red line indicates the damaged area after the polarization test.

Figure 6 shows SEM images of the Mg–Al LDH films prepared by (Figure 6a) steam coating at 140 °C for 12 h using 1.0 M Mg(NO_3_)_2_ aqueous solution, followed by immersion in 1.0 M aqueous solutions of (Figure 6b) Na_2_MoO_4_·2H_2_O, (Figure 6c) Na_3_VO_4_, and (Figure 6d) H_3_PO_4_, and elemental (Figure 6e) Mg and (Figure 6f) Mo mapping images of the defect part on the Mg–Al LDH film intercalated with MoO_4_^2−^ (film (Figure 6b)) after the polarization test. In Figure 6a,c,d, damage to the film was observed, likely resulting from pitting corrosion during the polarization test, as indicated by the increase in current density in the anodic region of the polarization curves. This pitting could have led to the exposure of the substrate surface to the NaCl aqueous solution, resulting in corrosion. Conversely, in film (Figure 6b), the presence of elemental Mo was confirmed, which could derive from MoO_3_ [32]. The proposed mechanism for MoO_3_ formation is initiated by the local acidification that accompanies pitting corrosion on the ADC12 alloy surface during immersion in a NaCl solution. Pitting corrosion leads to the hydrolysis of Al^3+^ ions within the pit cavity, which generates protons (H^+^) and lowers the local pH [33]:

Al^3+^ + 3H_2_O → Al(OH)_3_ + 3H^+^ (1)

The MoO_4_^2−^ ions, released from the interlayer of the LDH film, react with the generated protons (Equation (1)) to form the heptamolybdate ion (Mo_7_O_24_^6−^) [34]:

7MoO_4_^2−^ + 8H^+^ → Mo_7_O_24_^6−^ + 4H_2_O (2)

Subsequently, the Mo_7_O_24_^6−^ ions further react with protons to form the protective MoO_3_ film [35]:

6H^+^ + Mo_7_O_24_^6−^ → 7MoO_3_ + 3H_2_O (3)

The MoO_4_^2−^ ions released from the LDH film’s interlayer are deposited specifically on the damaged areas of the film as a self-healing layer via the sequential reactions outlined in Equation (1) through Equation (3). The released MoO_4_^2−^ first polymerizes into Mo_7_O_24_^6−^ ions, which then react with the locally produced protons to form the MoO_3_ film. Consequently, the MoO_3_ film does not form on the undamaged LDH surface, but selectively covers the damaged region, enabling the self-healing process as the reaction progresses in Equations (2) and (3) [32]. Thus, the MoO_3_ has been deposited on the damaged area of the Mg–Al LDH film because elemental Mo is localized in regions of low Mg concentration, as displayed in images Figure 6e,f. This deposition of MoO_3_ is inferred to have inhibited further corrosion reactions in the damaged area of the Mg–Al LDH film, thereby forming a stable passive region in the anodic region of the polarization curve.

**Figure 7:**
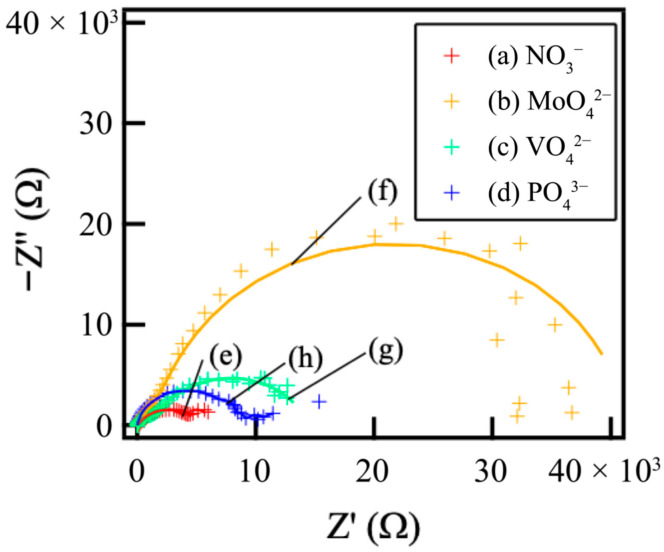
Nyquist plots of films prepared on ADC12 substrates via (a) steam coating at 140 °C for 12 h using 1.0 M Mg(NO_3_)_2_ aqueous solution, followed by immersion in 1.0 M aqueous solutions of (b) Na_2_MoO_4_·2H_2_O, (c) Na_3_VO_4,_ and (d) H_3_PO_4_, and fitting results of Nyquist plots of films prepared on ADC12 substrates via (e) steam coating at 140 °C for 12 h using 1.0 M Mg(NO_3_)_2_ aqueous solution, followed by immersion in 1.0 M aqueous solutions of (f) Na_2_MoO_4_·2H_2_O, (g) Na_3_VO_4,_ and (h) H_3_PO_4_.

Figure 7 shows the Nyquist plots of the films prepared on ADC12 substrates via (a) steam coating at 140 °C for 12 h using 1.0 M Mg(NO_3_)_2_ aqueous solution, followed by immersion in 1.0 M aqueous solutions of (b) Na_2_MoO_4_·2H_2_O, (c) Na_3_VO_4,_ and (d) H_3_PO_4_. As none of the samples yielded an ideal semicircle in the Nyquist plots, a constant phase element (CPE) was employed instead of an ideal capacitor to model the non-ideal capacitive response accurately. This deviation from ideal behavior is primarily ascribed to the film’s roughness and its inherent inhomogeneity [36]. The CPE is a special element whose value is a function of the angular frequency ω and whose phase is independent of the frequency. Its admittance (Y) and impedance (Z) are described as follows:

Y_CPE_ = Y_0_(iω)^n^ (4)

Z_CPE_ = 1/Y_0_(iω)^−n^ (5)

where Y_0_ is the magnitude of the CPE, ω is the angular frequency, and n is the exponential term of the CPE. Coated metal systems typically involve two distinct interfaces: the electrolyte/film interface and the film/substrate interface. Consequently, two time constants were necessary for the comprehensive evaluation. The first constant corresponds to the electrolyte/film interface, and the second to the film/metal interface [37,38]. Based on these electrochemical considerations, an appropriate equivalent circuit model was developed for the subsequent fitting analysis.

**Figure 8:**
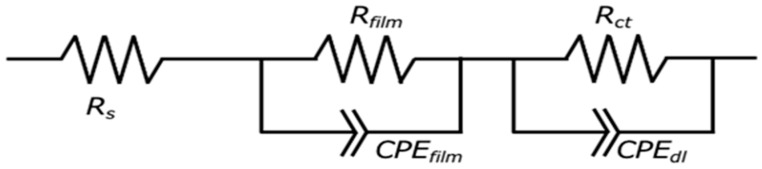
The equivalent circuit model for the LDH film. *R_s_*, *R_film_*, *CPE_film_*, *R_ct_*, and *CPE_dl_* show solution resistance, resistance of the film, substitute for the capacitor of the film, charge transfer resistance, and the capacitor of the electric double layer between the film and the substrates, respectively.

Figure 8 shows the equivalent circuit model for the Mg–Al LDH films. In the models shown in Figure 8, *R_s_*, *R_film_*, *CPE_film_*, *R_ct_*, and *CPE_dl_* show solution resistance, resistance of the film, capacitance of the film, charge transfer resistance, and capacitance of the electric double layer between the film and the substrates, respectively. The capacitive loops observed in Figure 7 are interpreted as the electrical double layer capacitance (*CPE_dl_*) in parallel with the charge transfer resistance (*R_ct_*). The *CPE_film_* is typically assigned to the capacitance originating from the surface film, which is influenced by various factors, including film thickness and defect structure. The *R_ct_* element, placed in parallel with *CPE_dl_*, represents the impedance associated with the reaction occurring at the film/substrate interface. The developed equivalent circuit model (Figure 8) was validated by the excellent fitting quality obtained for all Nyquist plots (Figure 7), characterized by small chi-squared (χ^2^) values (χ^2^ < 10^−4^ for all systems). This two-time-constant model effectively captures the distinct, depressed semicircles observed in the plots, corresponding to the high-frequency response (CPE_film_/R_film_) and the low-frequency response (CPE_dl_/R_ct_). Specifically, the film capacitance (CPE_film_) dominates the high-frequency domain, while the charge transfer process (R_ct_ and CPE_dl_) dictates the low-frequency behavior, demonstrating the appropriateness of the chosen circuit for analyzing the double-layered corrosion protection mechanism. The EIS simulation results of the films prepared on ADC12 substrates via (e) steam coating at 140 °C for 12 h using 1.0 M Mg(NO_3_)_2_ aqueous solution, followed by immersion in 1.0 M aqueous solutions of (f) Na_2_MoO_4_·2H_2_O, (g) Na_3_VO_4,_ and (h) H_3_PO_4_ are listed in Table 3. The *R_film_* values were observed to increase in the following order: MoO_4_^2−^ > VO_4_^3−^ > PO_4_^3−^ > NO_3_^−^. This trend aligns with the results of the polarization tests. As the thickness of each coating exhibited almost no variation, the effect of film thickness on the *R_film_* value is insignificant. Consequently, this value is considered to be largely dependent on the type of interlayer anion species. The Mg–Al LDH film, including NO_3_^−^, showed the smallest *R_film_* value among all samples. This can be explained by the fact that the Mg–Al LDH film containing NO_3_^−^ is more susceptible to corrosion factors such as Cl^−^, as the valence of NO_3_^−^ is lower than that of the other anions (MoO_4_^2−^, VO_4_^3−^, and PO_4_^3−^). Additionally, the stability of the anions between the layers of the LDH increases with higher valence. The Mg–Al LDH film with NO_3_^−^ may also be porous, indicated by its large *CPE_film_* value. Consequently, it is inferred that the structure of this film is non-uniform and defective, making it more permeable to the electrolyte. Conversely, the *R_film_* value of the Mg–Al LDH film intercalated with MoO_4_^2−^ was higher than that of the films containing VO_4_^3−^and PO_4_^3−^. This improvement is attributed to the fact that defects in the film were covered with MoO_3_, as shown in Figure 6b. The presence of the MoO_3_ layer significantly enhanced the corrosion resistance of the Mg–Al LDH film incorporating MoO_4_^2−^ by preventing corrosive electrolytes from reaching the substrate surface. The calculated CPE_film_ values are inversely related to the structural quality observed in the SEM images (Figure 2). For instance, the large CPE_film_ value obtained for the NO_3_^−^ intercalated film is consistent with the loose and porous structure shown in Figure 2c. Conversely, the significantly smaller CPE_film_ value for the MoO_4_^2−^ system (Table 3) reflects the highly dense and protective MoO_3_ layer formed at the defect sites (as discussed with Figure 6b), demonstrating a direct link between the electrochemical data and the observed morphology. The formation of a double-layered structure has been demonstrated to further enhance corrosion resistance [39,40,41]. Correspondingly, cross-sectional SEM observations of the fabricated LDH films revealed distinct top and bottom layers, strongly suggesting that this double-layered configuration may contribute to the improved corrosion resistance. Also, the corrosion resistance of the Mg–Al LDH films, including VO_4_^3−^ and PO_4_^3−^, was improved. This is because anions with higher valence exert stronger electrostatic interactions with the positive charges between the LDH layers, making them more stable within the layers. Cyclic potentiodynamic polarization test results showed that the Mg–Al LDH film intercalated with VO_4_^3−^ had higher corrosion resistance than that with PO_4_^3−^. Both *CPE_film_* and *CPE_dl_* values for the Mg–Al LDH film intercalated with VO_4_^3−^ were smaller than those of the Mg–Al LDH film intercalated with PO_4_^3−^, indicating that the denseness of the Mg–Al LDH film including VO_4_^3−^ was higher than that including PO_4_^3−^. This superior denseness in the VO_4_^3−^ intercalated film compared to the PO_4_^3−^ film is likely due to the specific size and geometry of the VO_4_^3−^ anion, which may provide better layer packing and fewer structural defects within the LDH interlayer space, thereby reducing the ionic permeability of the electrolyte. Thus, it is inferred that the corrosion reaction at the interface between the electrolyte and the Mg–Al LDH film with VO_4_^3−^ is less likely to occur than in the case of the film with PO_4_^3−^.

## 4. Conclusions

Mg–Al-layered double hydroxide (LDH) films, incorporating anions such as NO_3_^−^, MoO_4_^2−^, VO_4_^3−^, and PO_4_^3−^, were successfully prepared on ADC12 substrates using steam coating and immersion processes. These LDH films were characterized using XRD, SEM, FT-IR, and electrochemical measurements. XRD analyses revealed that the film prepared by steam coating had a mixed crystal structure composed of nitrate-type Mg–Al LDH and AlO(OH), and subsequent immersion formed a mixed film of Mg–Al LDH intercalated with MoO_4_^2−^, VO_4_^3−^, PO_4_^3−,^ and AlO(OH). SEM observations showed that all films developed petal-like structures, with estimated thicknesses of (a) 6.9 μm, (b) 7.3 μm, (c) 7.3 μm, and (d) 7.2 μm, respectively. XRD patterns and FT-IR spectra revealed that MoO_4_^2−^, VO_4_^3−^, and PO_4_^3−^ could be intercalated between interlayers of Mg–Al LDH by intercalation of nitrate acid-type Mg–Al LDH. We evaluated the corrosion resistance of Al–Si–Cu alloys coated with these films and found that the sample with the highest corrosion resistance was the Mg–Al LDH film, which had been prepared by steam coating at 140 °C for 12 h and subsequently immersed in a 1.0 M Na_2_MoO_4_·2H_2_O solution for 1 h. This film is the one intercalated with MoO_4_^2−^.

Given the increasing importance of environmental issues globally and the rising demand for aluminum alloys due to their lightweight, high productivity, and recyclability, we believe that the steam coating technology presented here effectively enhances the corrosion resistance of ADC12 alloy.

## Figures and Tables

**Table 1:** Chemical composition of ADC12 substrate (wt.%).

Si	Cu	Mg	Zn	Fe	Mn	Ni	Al
11.38	1.58	0.26	0.72	0.87	0.16	0.10	Bal.

**Table 2:** Atomic compositions of the Mg-Al LDH films on ADC12 substrates, as determined by EDS. The films were prepared by (a) steam coating at 140 °C for 12 h using 1.0 M Mg(NO_3_)_2_ aqueous solution, followed by immersion in 1.0 M aqueous solutions of (b) Na_2_MoO_4_·2H_2_O, (c) Na_3_VO_4,_ and (d) H_3_PO_4_ (at.%).

Samples	N	O	Mg	Al	Mo	V	P
(a) NO_3_^−^	2.6	63.5	21.7	12.2	ND	ND	ND
(b) MoO_4_^2−^	ND	68.4	11.4	9.2	5.7	ND	ND
(c) VO_4_^3−^	1.6	63.6	16.1	9.2	ND	4.7	ND
(d) PO_4_^3−^	ND	62.0	13.6	12.3	ND	ND	4.4

**Table 3:** EIS simulation results of (a) steam coating at 140 °C for 12 h using 1.0 M Mg(NO_3_)_2_ aqueous solution, followed by immersion in 1.0 M aqueous solutions of (b) Na_2_MoO_4_·2H_2_O, (c) Na_3_VO_4,_ and (d) H_3_PO_4_.

Samples	*R_s_* (Ω·cm^2^)	*R_film_* (Ω·cm^2^)	*CPE_film_* (Ω^−1^·s^n^·cm^−2^)	*R_ct_* (Ω·cm^2^)	*CPE_dl_* (Ω^−1^·s^n^·cm^−2^)
(a) NO_3_^−^	17.1	4.16 × 10^3^	1.52× 10^−4^	6.69 × 10^2^	4.37 × 10^−2^
(b) MoO_4_^2−^	16.7	3.89 × 10^4^	3.52 × 10^−5^	2.35 × 10^3^	4.17 × 10^−5^
(c) VO_4_^3−^	15.1	1.32 × 10^4^	1.41 × 10^−4^	1.36 × 10^3^	3.02 × 10^−5^
(d) PO_4_^3−^	20.4	9.95 × 10^3^	5.10 × 10^−4^	3.68 × 10^3^	2.92 × 10^−4^

## Data Availability

The original contributions presented in this study are included in the article. Further inquiries can be directed to the corresponding author.
